# Fat, fibrosis, and the future: navigating the maze of MASLD/MASH

**DOI:** 10.1172/JCI186418

**Published:** 2025-04-01

**Authors:** Scott L. Friedman

**Affiliations:** Icahn School of Medicine at Mount Sinai, New York, New York, USA.

The worldwide challenge of metabolic dysfunction–associated steatotic liver disease (MASLD) and its more advanced form, metabolic dysfunction associated steatohepatitis (MASH), has crept into our world. Previously referred to as NAFLD and NASH, respectively, these illnesses affect 30%–40% of the world’s population and are not confined to developing countries, rather affecting all regions except sub-Saharan Africa (1, 2). In patients with type 2 diabetes, the prevalence of MASLD is estimated at approximately 65% (3), and even young adults are at risk for significant fibrosis (4). The burden of MASLD in the US is predicted to rise significantly in the next 30 years unless effective therapies are established (5). The increasing worldwide prevalence, its economic impact, and the adverse sequelae of MASLD and MASH (summarized briefly in Figure 1) have galvanized efforts to fully elucidate pathobiology and refine treatments, many of which will be explored in this series of Reviews.

The new nomenclature of MASLD and MASH emerged in 2023 from a deliberative process that was intended to remove the stigma associated with linking the illnesses to alcohol and alcohol misuse as well as to allow more accuracy in defining the disease spectrum and cofactors (6).

MASLD is defined as the presence of 5% or more steatosis in the liver in the absence of significant alcohol ingestion and is associated with metabolic risk factors, including overweight, type 2 diabetes, and associated conditions that include hypertension, hyperlipidemia, or insulin resistance. In up to 20% of individuals, MASLD can progress to MASH. MASH is a serious concern because it is marked by inflammation and fibrosis, which confer a progressive risk of liver dysfunction, failure, and primary liver cancer, or hepatocellular carcinoma (HCC). It’s critical to point out that while fat and inflammation are prominent features of MASH, fibrosis is the sole histologic feature of MASH that correlates with clinical outcomes, and thus focusing on this feature of the disease remains a high priority, especially in patients with advanced fibrosis and cirrhosis.

The public health and economic impacts of the MASLD/MASH disease spectrum are staggering. Healthcare costs in patients with MASLD are up to twice those of healthy counterparts, especially in those with more advanced stages with MASH (7, 8). A 2023 study modeling the economic and clinical implications of MASLD/MASH projected that the MASH-attributable healthcare cost per patient is expected to rise from $3,636 to $6,968 by 2039, primarily as a result of increasing costs of caring for patients with advanced disease (7).

MASLD/MASH are clearly recognized as hepatic manifestations of a systemic disease often referred to as metabolic syndrome, which is associated with heightened cardiovascular morbidity and mortality, as well as a host of nonliver manifestations. Collectively, this constellation of abnormalities imposes a substantial threat to the health-related quality of life, including a high symptom burden, which impacts physical and mental health and reduces productivity (9). Family history can be a significant risk factor, with up to 15% prevalence of advanced fibrosis among first-degree relatives of MASLD patients with advanced fibrosis (10). From these significant risks has emerged a new holistic focus on metabolic health, with growing awareness of its close link to liver health.

Always lurking in advanced MASH is the risk of HCC (11). HCC in MASH presents unique problems. First, there is a higher risk that cancer will develop before patients are diagnosed with cirrhosis, which is when screening programs are typically implemented for other chronic fibrotic liver diseases. The result is a higher likelihood of diagnosing HCC in MASH at advanced stages when treatment options are limited and cure is unlikely. Second, MASH-related HCC may be resistant to the checkpoint therapies that represent a recent breakthrough in treating cancer (12). Thus, we need to learn more about the unique immunologic microenvironment that promotes HCC in MASH (11).

Against this backdrop is a recent surge of revolutionary antiobesity therapies targeting incretin signaling (e.g., the GLP-1 agonist semaglutide) and sodium-glucose cotransporters (sodium-glucose cotransporter 2 [SGLT2] inhibitors). Although these therapies can elicit significant reductions in weight and liver fat, it is too early to determine whether they will impact the prevalence and natural history of MASLD and MASH. Early indications are that while weight loss can promote improvement in MASH, it does not guarantee it. This observation raises the key question of disease heterogeneity. While patients with MASH have shared features of histology, there may be subtypes of disease emerging in several recent studies, wherein key disease drivers — and therefore therapeutic targets — may differ across the affected population. This heterogeneity (13–15) may explain why the same drugs do not work in all patients, heightening the need to clarify risk factors, disease drivers, and predictors of drug response among patients.

With only one drug approved to date for MASH, the unmet need for progress is clear, and challenges abound. Why have MASLD and MASH appeared only in the past approximately 25 years? How can we accurately diagnose and stage the disease without relying upon an invasive liver biopsy? Why do only some patients respond to therapies?

This brief summary highlights many of the key unmet needs in understanding and treating MASLD/MASH, including new insights into pathogenesis and risk and prospects for novel therapies for this expanding public health threat. These questions will be tackled by a series of expert-led Review articles authored by leading investigators representing the spectrum of the disease, from basic investigation to clinical trials. I hope these articles are informative and thought provoking, providing both answers and questions as we seek to unravel a complex challenge through great science, imagination, and grit.

## Figures and Tables

**Figure 1:**
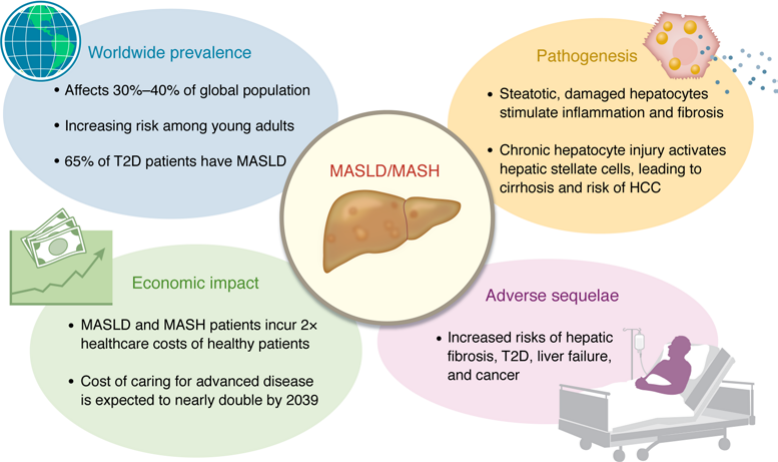
Concerns about the increasing worldwide prevalence, economic impact, and serious adverse sequelae of MASLD and MASH draw attention to its pathogenesis and treatment options.

## Version 1 04/01/2025

Electronic publication
